# Strengthening Antenatal Care towards a Salutogenic Approach: A Meta-Ethnography

**DOI:** 10.3390/ijerph18105168

**Published:** 2021-05-13

**Authors:** Kristiina Heinonen

**Affiliations:** 1 Health Care and Health Promotion, Metropolia University of Applied Sciences, P.O. Box 4000, FI-00079 Helsinki, Finland; kristiina.heinonen@metropolia.fi; 2 Department of Nursing Science, University of Eastern Finland, Yliopistonranta 1, FI-70210 Kuopio, Finland

**Keywords:** antenatal care, midwife, meta-ethnography, meta-synthesis, nurse, prenatal care, qualitative research, salutogenic approach

## Abstract

The aim was to explore how midwives, public health nurses and nurses view caring in antenatal care (ANC) as provided for mothers and fathers/partners. Based on Noblit and Hare (1988), meta-ethnography was used to address meaning by synthesizing knowledge and understanding inductively through selected qualitative studies (*n* = 16). Four core themes were identified: (1) supporting the parents to awaken to parenthood and creating a firm foundation for early parenting and their new life situation; (2) guiding parents on the path to parenthood and new responsibility; (3) ensuring normality and the bond between baby and parents while protecting life; and (4) promoting the health and wellbeing of the family today and in the future. The overarching theme can be expressed as “helping the woman and her partner prepare for their new life with the child by providing individualized, shared care, firmly grounded and with a view of the future”. Caring in antenatal care (ANC) is being totally present, listening and using multidimensional professional competence but also being open-minded to new aspects and knowledge. The health promotion and positive health aspects should be considered an important part of supporting parents and the whole family now and in the future. A more conscious salutogenic approach to ANC would lead to more favorable results and could be a fruitful research topic in the future. There is a need to provide midwives/nurses with enough time to allow them to concentrate on specific needs and support for different kind of families in ANC but also training for midwives to make them more familiar with online and other options.

## 1. Introduction

People experience different kinds of transitional phases in their lives—related to developmental changes, changes in certain life situations, and health and illness. Pregnancy and the birth of a child signify a transitional phase in the life of the parents [1]. Antenatal care (ANC) provides support and guidance in the transition to parenthood, as provided by midwives, public health nurses and nurses, depending on the country. ANC is guided by international and national codes of ethics for midwives and nurses [2,3]. 

In ANC, the concept of the person focuses on the expectant mother and family. Emphasis on caring and interaction with women has been described as a women-centered philosophy [4], women-centered care [5], connected care [6] and holistic care [7]. Midwives and nurses providing ANC come into contact with women from different backgrounds, different cultures and in different family situations such as two- and single-parent families, multiple-birth families, families with migrant backgrounds, bicultural families, blended families, adoptive families and rainbow families. [8].

## 2. Background

Although the quality of ANC (antenatal care) varies globally, the World Health Organization (WHO) envisages a world where “every pregnant woman and newborn receives quality care throughout the pregnancy, childbirth and the postnatal period”. The WHO (2016) recommendations on ANC for a positive pregnancy experience include nutritional interventions, maternal assessment, preventive measures, interventions for common physiological symptoms, and health system interventions to improve the utilization and quality of antenatal care. In addition, they recommend risk identification, prevention and management of pregnancy-related or concurrent diseases, health education and health promotion. Evidence-based knowledge and practice is what is needed [9]. ANC has a strong focus on the woman and child, but many studies have found that fathers/partners would like to be involved more and support women during pregnancy, an aspect of care that has been lacking. [10,11,12,13,14,15,16,17]. Fathers are also concerned about the safety of the women and babies during labor [18].

Provision of effective maternity care is a vital global policy goal [19]. Parenting education supports the transition to parenthood by allowing couples to reflect and talk about parenthood during pregnancy [20]. Ferguson et al. [21] show that women with a strong sense of coherence have a positive, relaxed and baby-focused attitude, while a weak sense of coherence is expressed through a negative, worried and labor-focused attitude towards labor and birth [21]. Ferguson et al. [22] have shown that satisfaction with birth raised women’s sense of coherence, which is an important aim of antenatal education. An increased understanding of the effect of sense of coherence on women’s attitudes to childbearing has also led to the development of salutogenic antenatal education [22].

The International Confederation of Midwives (ICM) promotes the midwifery model of care based on respect for human dignity, compassion and the promotion of human rights for all persons. Women-centered care also includes autonomy and advocacy [3]. The ANC model is to provide pregnant women with respectful, individualized, person-centered care at every contact, with implementation of effective clinical practices (interventions and tests), and provision of relevant and timely information, and psychosocial and emotional support, by practitioners with good clinical and interpersonal skills within a well-functioning health system. It is also important to listen to parents and help them find their own resources, while promoting healthy habits and the ability to make better choices for the child’s and whole family’s wellbeing. This involves supporting the couple’s relationship and encouraging them to seek peer support and ask others for help [8].

The relationship between healthcare providers and pregnant women plays a role in their engagement in prenatal care [5]. The quality of care rests on the centrality of a meaningful relationship with ANC clients. It helps women accept guidance and health-related advice and follow recommendations. Such a relationship fosters trust, comfort and a reduction in anxiety for clients. It also means involving women as active partners in their care, e.g., by giving them responsibility for routine aspects of care and letting them participate in decision-making, which also helps care providers when they need to be more directive [5]. Mellor, Payne and McAra-Couper [23] found that mental health services were lacking when midwives needed to refer pregnant women with mental health issues, and midwives routinely assessed women’s mental health during antenatal care in informal and not necessarily explicit ways. They were concerned about the introduction of routine universal antenatal screening without the availability of appropriate services for women with mental health issues such as anxiety [23]. There is a need for relaxation training during pregnancy that promotes the wellbeing of mother, fetus and baby [24].

All the women feared the pain of childbirth, and more than half that they would not to be able to achieve a normal birth, or that the baby would be injured during the birth process. The most common fears for men were that their partner’s mental health would suffer as a result of a traumatic birth, that they would be unable to provide adequate support during labor or that the baby would be injured. “Riskiness”, “ways of coping” and “being a good parent” are related to the comprehensibility, manageability and meaningfulness dimension of sense of coherence [18]. Rollans, Schmied, Kemp and Meade [25] show that the style or approach a midwife takes to psychosocial assessment is important as this may impact on a woman’s comfort in disclosing her concerns and on her relationship with the maternity service. Some of the midwives followed a structured format tending to deliver the questions in a directive manner, whereas others appeared more flexible in their approach and delivery of sensitive questions. Regarding the patient–nurse relationship, nurses practicing connected care reported developing a stronger relationship with their patients, based on more interactions, and felt able to give better personalized care [25]. Good communication, e.g., giving women time to answer questions, listening to their answers and checking for accuracy in understanding, helps to normalize their pregnancy [6]. Wikberg, Eriksson and Bondas [26] found that the care of immigrant new mothers in Finland was influenced by the cultural background of the mother as well as the Finnish maternity care culture. The stereotypes and ethnocentric views of some nurses negatively influenced the experience of maternity care for some mothers. However, despite the differences in the expectations of the mothers and their experience of Finnish maternity care, female nurses were seen as professional friends, and conflicts were resolved, which promoted caring [26]. Midwives encounter a variety of challenges, such as tailoring care to individual needs, dealing with stereotypes, addressing varied levels of health literacy, overcoming communication barriers and enabling partner involvement [27].

In 1998, Olsson, Jansson and Norberg pointed out that ANC focuses more on the mother than the father/partner [28]. In the meta-synthesis by Chin et al. [14], some fathers described feelings of being outsiders [10,15], distant [11,29], overlooked in antenatal classes [13] and excluded from appointments and antenatal classes [12]. Steen et al. [15] show that fathers feel themselves to be a “partner and parent”, but they experience being “not-patient and not-visitor” in their encounters with maternity care services [15]. Johansson, Fenwick and Premberg [16] reported that expectant fathers described a strong desire to participate and be actively involved in their partner’s labor, but they still had overwhelming feelings of inadequacy in their ability to support their partner. For fathers, participating and being present was a unique and exclusive opportunity to “bond” with their newborn child. Being prepared and receiving support were essential elements of positive experience as well as contributing to their ability to adequately support the woman [16]. Fathers and public health nurses described different ways of being a father: bystander, supporter of spouse, partner and head of family [30]. Wilmore et al. [31] found that midwives and other antenatal staff use a range of strategies in their services. For some fathers, the sense of connection and involvement in the pregnancy increased as the pregnancy progressed, which appeared to be linked to men’s engagement in various activities during ANC. Healthcare professionals involved with parents-to-be during pregnancy should actively encourage men to engage in different kinds of activities [14]. Men who attended classes were better prepared to support the woman and cope with labor and any critical events during the process. Positive, respectful behavior and language by professionals impacted greatly on men’s sense of involvement [16].

There is a need for individual but flexible and supportive ANC for parents having a baby. Expectant parents are hoping for care and a healthy baby but also for a lot of support for parenting. Today, online support is also important, a need that has increased during the pandemic of 2020. There are various studies presenting different views of antenatal care, but a holistic understanding of what caring means in such care is lacking. In the current meta-synthesis of previous qualitative studies, the focus was on deepening the understanding of caring in ANC and the relationship with the woman, her partner and family from the perspective of midwives and nurses. Meta-synthesis may reveal new insights and different views on providing ANC [32,33]. The purpose of synthesizing the findings of previous research was to gain a holistic understanding of caring in ANC and disclose the relationship between parents and social and health care professionals.

### 2.1. Theoretical Perspective

The salutogenic model [34] offers a positive way of promoting the health and wellbeing of parents and their child during pregnancy and birth and in the future. A supportive culture provides people with the resources needed to perceive life as comprehensible, manageable and meaningful, all three elements being integral to the sense of coherence [34,35]. Given the different backgrounds, resources, life course stress exposures and life experiences of those in the transition to parenthood, sense of coherence—comprehensibility (cognitive), manageability (behavioral) and meaningfulness (motivational)—provides a universal basis for midwives and nurses to support parents and promote the whole family’s health and wellbeing. 

Salutogenesis offers midwives an opportunity to positively influence public health by empowering parents to confidently take control of their birthing and mothering experience. It is also a way of guiding women towards a goal of health and wellbeing rather than avoiding disease [36]. Sociodemographic background, psychological and birth-related factors and availability of social support affect sense of coherence among parents, which is positively correlated to the quality of the relationship, satisfaction with perceived support, psychological wellbeing and overall birth experience [37]. According to Vinje, Ausland and Langeland [38], health professionals’ “salutogenic capacity” is their degree of skill to help a person or group examine, mobilize and deploy sufficient resources to achieve a shift towards the experience of good health and wellbeing [38]. This is needed in different life situations during the course of life.

### 2.2. The Review

#### 2.2.1. The Aim

The purpose of this meta-ethnography was to explore how midwives, public health nurses and nurses view caring in antenatal care as provided for mothers and fathers/partners.

#### 2.2.2. Design

In accordance with Noblit and Hare [32], meta-ethnography was chosen to synthesise knowledge from qualitative studies. This approach is able to address meaning by synthesizing knowledge and understanding inductively through selected studies and showing relevance in context. It consists of seven phases, which are usually overlapping instead of linear: (1) getting started and identifying the topic; (2) deciding what is relevant to the initial interest; (3) reading the studies; (4) determining how the studies are related; (5) translating the studies into one another; (6) synthesizing translations and (7) expressing the synthesis [32,39]. The eMERGe meta-ethnography reporting guidelines were used to increase transparency, strengthen trustworthiness and credibility and help identify useful findings and positive interventions [40,41] (Table 1).

#### 2.2.3. Method

Records were identified through database searching using Cinahl, Copus, Medline, ProQuest/Health, Psycinfo, PubMed and Wiley. Literature searches were conducted in April and May 2018, followed by an updated later. Manual searches were also performed in various scientific journals such as Birth, BMC Pregnancy and Childbirth, International Journal of Childbirth, Journal of Advanced Nursing, Journal of Midwifery and Women’s Health, Journal of Obstetrics, Gynecology and Neonatal Nursing, Sexual and Reproductive Health, Qualitative Health Research, Women and Birth.

The selected studies had to meet the following inclusion criteria: qualitative methods (mixed methods were included with qualitative methods); focus on normal pregnancy without complications, based on caring in ANC provided by midwives, public health nurses and nurses for parents; the views of midwives, public health nurses and nurses (different views [mothers, fathers/partners, general practitioners, obstetricians] were included when it was possible to separate the perspective of the midwife and nurse); published in English or a Nordic language in peer-reviewed scientific journals. 

Collaboration was employed in excluding articles based on reading of the title, abstract and/or the full text such as the article were outside the research focus (focus on the organization of delivery and utilization of antenatal care; ICT (information and communication technologies) communication technology/digitalization; the role of midwives, their knowledge needs and the workplace culture; care plans and documentation; cultural issues; screenings; care in relation to issues encountered in ANC such as violence, obesity, diet counselling, asthma, depression, smoking and antenatal care for adolescent mothers and for alcohol and other drug users, or poverty). Furthermore, also excluded were quantitative studies, theoretical papers, reviews, masters’ theses, and dissertations. Search filters limiting publication year were not used so as not to exclude relevant older studies.

**Figure 1:**
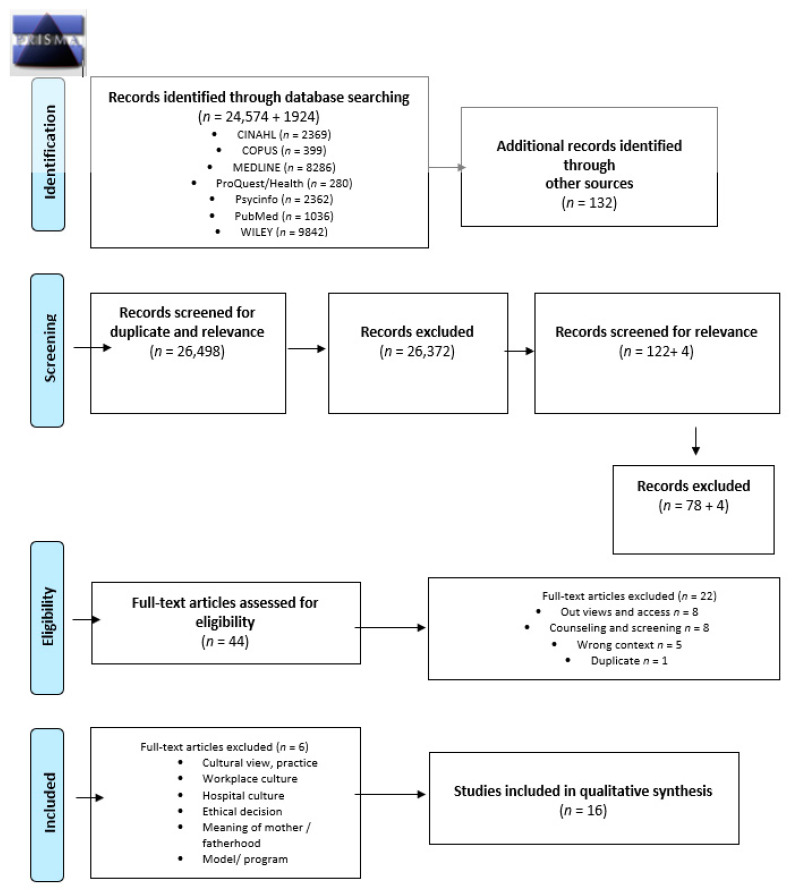
Prisma (2019) Flow Diagram. From: Moher, D.; Liberati, A., Tetzlaff, J., Altman, D.G., the PRISMA Group. Preferred Reporting Items for Systematic Reviews and MetaAnalyses: The PRISMA Statement. *PLoS Med* **2009**, *6*, e1000097. doi:10.1371/journal.pmed1000097. Available online: www.prisma-statement.org (accessed on 10 May 2021) [52].

The keywords identify studies focusing on the right context, caring in ANC and social and health care professionals such as midwives and public health nurses (Table 2). The total search brings forth *n* = 24,574 articles from databases and *n* = 132 articles for different journals (Figure 1). The responsibility for conducting the literature searches was shared between the authors, and experienced librarians from two different countries assisted in finding relevant key terms and performing the searches. This meta-ethnography concentrates on the views of midwives, public health nurses and nurses working in ANC. Later references are only to midwives and nurses. Included were sixteen nursing studies (*n* = 16): Ahlden et al., [20], Andersson et al., [42], Aquino et al., [7], Baron et al., [6], Browne et al., [17], Dalton et al., [43], Dove and Muir-Cocrane, [44] Goodwin et al., [45], McCourt [46], Olsson et al., [47], Proctor [48], Rominov et al., [49], Saftner et al., [50], Sword et al., [5], Withford et al., [51] and ja Wright et al., [4]. The articles were published in English in peer-reviewed journals between 1996 and 2018. Five of the studies were conducted in Australia and in the UK, three in Sweden, two in the US and one in Canada. Altogether, the papers involved midwives *n* = 371 and certified Nurse-Midwives and nurses (practiced in obstetrics/maternity care minimum 2 years), *n* = 23 (total *n* = 394). Study participants also included maternity service staff, maternity care providers or maternity/prenatal care providers such as obstetricians, obstetrician-gynecologists, physicians, family physicians, general practitioners and grades (consultants and specialist trainee obstetricians). 

#### 2.2.4. Quality Appraisal

The papers to be included were chosen collaboratively and the Critical Appraisal Skills Programme (CASP) was used for help. The checklist served as a systematic reminder of issues related to quality including study aim, methodology, recruitment strategy, data collection, reflexivity, ethical issues, data analysis, statement of findings and study value. The author assessed all articles that were eligible for inclusion, and the other shared the articles between them to ensure that two authors assessed all the articles independently. Final agreement on inclusion was reached in a Skype meeting. Quality assessment of selected studies was tabulated (Table 3).

#### 2.2.5. Data Extraction and Synthesis

The analysis process started with all the researchers deciding on the focus of the synthesis and continued with a thorough literature search, identifying and selecting relevant studies.

The next phase involved paying closer attention to the details, identifying study characteristics and repeated reading of the studies. Creating a table with characteristics of selected studies and key findings helped to continue to the next phase. (Table 4) Following Noblit and Hare’s [32] guidelines for determining how the studies are related (Phase 4), we continued by immersing ourselves in the data and noting the interpretative metaphors and/or core concepts throughout the studies [32]. This process was performed by the researchers individually and in pairs and was facilitated by the use of lists, figures and tables to identify and compare the first- and second-order concepts across the studies. The findings were incorporated from the studies into one another by analogue translation to form new third-order refutational concepts. The analysis process was iterative and involved moving back and forth in the data, comparing and contrasting the findings from the individual studies and translating them into one another (Phase 5). The synthesis translation allowed themes to emerge and an overarching lines-of-argument model based on four core themes (Phase 6). The final stage (Phase 7) involved summarizing the main interpretive findings of the translation and synthesis [32]. The Grade Cerqual with themes helped to follow decisions during the research process and the results [39,53].

## 3. Results

Four core themes were identified: (1) supporting the parents to awaken to parenthood and creating a firm foundation for early parenting and their new situation; (2) guiding parents on the path to parenthood and new responsibility; (3) ensuring normality and the bond between baby and parents while protecting life; and (4) promoting the wellbeing and health of the family today and in the future. The overarching theme can be summarized as “helping the woman and her partner prepare for their new life with the child by providing individualized, shared care, firmly grounded and with a view of the future” (Table 5).

### 3.1. Supporting the Parents to Awaken to Parenthood and Creating a Firm Foundation for Early Parenting and Their New Situation

#### 3.1.1. Welcoming, Being Actively Present and Available

From the outset, it is important for the midwife to develop a good rapport with the mother as the basis for trust, and later, for reconciling non-verbal and verbal cues and the impact of the environment [17]. When meeting women, midwives try to create a positive atmosphere where the women feel welcomed and important. This begins with the greeting phase [4,49], and midwives also use humor and laughter to make a quick connection [17] while being calm and relaxed in their body language [5,17]. Midwives try not to look busy or show stress during meetings, but concentrate as much as possible on the women, not using the computer until after the appointment and showing that the visit is about the woman, not the midwife. Many things together help the midwife appear present and welcoming to the mother [17].

#### 3.1.2. Connecting and Creating a Relationship with Both Parents

Some of the midwives considered it important from the beginning to show interest in and connect with both parents, not only the women. This meant having the opportunity to prepare the parents together for their new life situation, as well as encouraging their involvement and choices. Midwives were supportive of women who chose to include significant others or other family members in their care [4,50], were willing and pleased to learn about the parents’ specific life [45,47], encouraged them to trust their own feelings and express their views and choices [42], supported the parents in preparing for parenthood through parenting education, and strengthened their confidence while reducing stress [20]. In the study by [6], healthcare providers reported using personal professional connections to obtain more information to help parents.

Actively connecting meant the midwife using eye contact, introducing herself to both parents, asking the father’s name and occupation, seeing them both as parents and encouraging their involvement and choices. “I am looking after you and your family today”, rather than “I am looking after her (and not you)” [49]. They considered the uniqueness of the expectant parents. It also meant emotional involvement, striving to connect with parents, showing confidence in their experiences, knowledge and ability, being open to their views and choices and showing willingness to support their ability to understand and meet the challenges they would face [47].

#### 3.1.3. Building Continuity and Trust in the Relationship between Midwife and Women

The continuity of the relationship means being available and supporting women in different situations during their pregnancy. Many studies report midwives referring to their continuing relationship over the period of pregnancy until after childbirth. [17,44,48,50]. This meant a childbearing woman receiving her perinatal care from a trusted midwife or a small team of midwives that had contact with the woman throughout the perinatal period [49]. In the study by [50] a relationship of trust between a woman and her provider was characterized by familiarity, “being on the same page”, positive encouragement, shared decision-making, and feeling safe and relaxed. Trust was central to women’s sense of safety; “… even if it is the first baby a woman knows that something is not quite right. It only takes me to say two words and they say ‘Yeah, I was already feeling that, let’s go’” [44].

Midwives used humor and laughter to help them connect with and focus on the women, to encourage conversation and their involvement in their own care. It was important for midwives to develop a constant relationship with the women [42,47] and to become more active in their care [17,47], which also strengthens the relationship. “I think that continuity is the biggest thing because you know what [the women] are worried about if you know them well. By the time they are having their tests they do share with you.” [17]. During antenatal care, continuity also meant supporting the lifelong relationship between parents and children [20] using well-developed skills to connect with women and a birth plan to support discussion [4]. A trusted relationship with providers facilitates the women’s care: “Well, I think if we have a longstanding relationship, it’s much easier to explain a little bit about how intense the whole process can be, how much they trust their nurse, their doula, their midwife, to give them good counsel about how they are really doing…” [50].

#### 3.1.4. Actively Getting to Know the Father/Partner to Encourage and Engage Them

From the beginning of the pregnancy, midwives found it important to encourage and engage fathers to be responsible parents. They looked for information to help them get to know the father/partner. Most studies place emphasis on women, but some studies present care models facilitating development of rapport with the family by enabling more contact, engagement and support for fathers. Midwives want to get to know the father, too, to encourage and involve him: “He is a part of this and I want to meet him…” Midwives prepared for appointments by reviewing case records, for example, allowing them to commence their consultations with a focus on the women and family without distraction [4]. They also gave their contact details and welcomed the fathers [49], requested to see them and showed interest in their feelings such as involvement, happiness and insecurity [42].

Being a midwife means actively engaging fathers, but also having a goal for this process as a professional. “Engaging fathers early helps them build confidence in their parenting abilities and their ability to support their partner” [5]. The midwives also emphasize the father’s/partner’s role during antenatal care. Fathers were welcomed, especially to the parenting groups, which also promotes the transition to parenthood: “… the men’s situation must be affirmed… we must involve them to a greater extent, they must not feel marginalized.” Most of the midwives found they had sufficient knowledge, skills and confidence for this [49].

There were also different views on fathers/partners being in antenatal care. In a study by Olsson et al. (1996), fathers were welcomed but were passive like strange visitors in a women’s world, feeling removed from what was being discussed [5,45,47,48]. Some of the midwives did not even mention the importance of involving women’s partners even though women themselves find it important. This meant women mostly reading and discussing leaflets with their partner at home [48].

#### 3.1.5. Recognizing the Importance of Language in Providing Support and Dealing with Challenges

Providing sensitive ANC involves making sure that the pregnant woman and her family understand what is discussed, what the midwife is doing and the instructions given, but also that there is enough time and space for the woman/partner to express themselves. This means using understandable language and giving clear information/instructions. There were cultural and linguistic challenges and a need for more individual understanding and care. In a study by [4], it was evident that communication was an integral part of how the midwives might support women, and the use of language was seen as an important tool to hear the woman’s voice.

In culturally different ANC settings, midwives encountered a variety of challenges and difficulties in providing individual care. [7,42,45,49] Sometimes it was also difficult to articulate cultural norms and beliefs with parents [7,49]: “… they may not understand the role of a midwife. It may be very doctor-orientated for a lot of women and they won’t expect the role of midwife to be so involved especially if there’s no complications… sometimes that’s a challenge explaining you know the role of midwife.” Different situations required different kinds of words and language use. Midwives are supportive by being aware of and encouraging in their language use and giving space to interact, supporting normality and understanding and choosing to reframe language and terminology of risk [4,17]. In the study by [17], some of the midwives wrote positive and supportive language messages for the women on their antenatal records.

Language depends on the cultural context and involves factors that may lead to barriers for midwives in providing support as part of their profession. Language is affected by relationships, including family relationships, culture and religion as well as differences in healthcare systems [45]. Language skills in English were limited and non-verbal communication was considered vital to care, such as explaining medical procedures and providing other maternity care information [7]. Language challenges led to difficulties in engaging with women and working with culturally diverse fathers [49]. Open-ended questions and attending to the women helped them articulate their thoughts and promoted a more supportive midwife-woman interaction: “Terri uses little jargon, interview very much a conversation, lots of eye contact and smiles… asks, ‘How are you feeling in yourself?’”.

The skills to attune their language differed between midwives. Some of them had problems or were unclear in referring to pregnant women in ANC, calling them “the patient/s, client/s, the woman and women” [4]. Sometimes midwives expected women to have the same understanding as them and noticed that the women’s cultural and/or regional practices were not supported within NHS (National Health Services) maternity care, leading to the need for sensitivity. The different expectations among women in ANC led to a great deal of dissatisfaction [7].

### 3.2. Guiding Parents on the Path to Parenthood and New Responsibility

#### 3.2.1. Listening to Each Person to Encourage Participation

An important part of antenatal care is treating women as individuals and enabling them to participate in their own care as much as possible. Midwives gave women space to voice their feelings and listened, helped and encouraged their participation, using the women’s own stories and experiences to communicate with them [4,17,44,46,51]. This meant being interested in the whole person and their life, not just measuring and controlling (fetal growth, blood pressure, vital checks) [17]. Midwives showed continuous interest in the women’s individual birth stories to identify and share their knowledge with them, preparing for birth and contextualizing individual risk by grounding a woman’s risk in her experience, past and present [44]. In the study by [6], connected care makes for more interactions and better personalized care and thus a stronger relationship with patients and better nurse–patient communication. Wright et al. [4] stresses effective woman-centered care where the rituals and practices that engage women together with knowledge of interpersonal skills are integral.

#### 3.2.2. Supporting Women’s Self-Determination

Midwives try to help women take part in their own antenatal care in various ways. In a study by [47], midwives shared a large amount of information with women, such as weight, blood pressure, urine analysis, and ultrasound examinations. They also provided information on emotional experiences and expectations of pregnancy, birth and parenthood, and the relationship between the couple and the expectant parents’ own parents [47]. Proctor [48] found also that some women preferred to defer to the professionals rather than be actively involved themselves in care decisions as many younger women were not used to making choices.

Midwives supported women’s self-determination by listening to and learning about their concerns [4], encouraging problem-solving and coping [17,44] and negotiating decision-making about care choices [44]. “… but I think if I don’t provide an opportunity for the woman to say she has a worry or a concern or something, then she might never say it.” Midwives also used gentle questioning techniques to enable them to tap into women’s wisdom as a way of problem-solving and coping [17]. There were three interactional styles used by midwives that differed in their effectiveness: professional (expert guidance), partnership (participative or collaborative) and disciplinary (expert surveillance) [46]. During caseload interviews and discussions, midwives really tried to get to know the women and offered them more information and the possibility to choose, “it is your choice”, “what we offer here”, “you can have it”, and control [46]. Responding to women’s individual preferences also meant using a personal birth plan, even though it was not always made available later [51]. It was also important to discuss past personal experiences, labor and birth to help women orient themselves with confidence to the new situation [50]. In the cultural context, there were challenges for midwives in harshly judging a woman’s decision-making if a positive relationship was not yet established between the midwife and the woman: “… if you haven’t built up a relationship with somebody during pregnancy… you tend to actually be very hard on some of the decisions they make, and I think we need to be honest about it.” [45].

In the study by [48], midwives recognized that it was not easy for all women to be actively involved in their care decisions, even though the opportunity was given and supported by midwives, and especially younger women preferred to defer to the professional’s advice.

#### 3.2.3. Building and Supporting the Confidence of Fathers/Partners 

Midwives found it important to actively involve fathers in ANC, which had a positive effect on the fathers and the family. The study by [49] found it was important to notice men’s level of receptiveness to perinatal services, which helps the process of engaging fathers and building their confidence. Midwives considered engaging fathers to be an important part of a midwife’s role, involving more contact with and support for fathers, and also allowing them to call the midwife if they had questions [49]. Midwives considered and showed interest in expectant fathers, addressing specific questions to them [47] and speaking to both new parents as a couple expecting a child and preparing for parenthood [49]. In some studies, midwives described the good goals of this process: “Engaging fathers early helps them build confidence in their parenting abilities and their ability to support their partner” [5]. “I feel strongly that the more a father is engaged with, the better the parent-bond will be with his baby” and “the dads that engage and want to engage, the relationship between mother and the father is usually much stronger and palpably so” [49]. It was also important to prepare parents for the labor and include the woman’s partner, family and friends in discussing the positive encouragement they could give her [50].

For various reasons, some of the studies found challenges in supporting fathers and/or building their confidence. Olsson et al. [47] observed that expectant fathers seemed like strange visitors in a women’s world, and problems arose in relation to midwives’ disregard of the fathers’ uniqueness. The fathers were lost because midwives did not mention the importance of involving women’s partners in care decisions [48].

With regard to different cultures, although family members were a source of support to women, midwives perceived this involvement as having a negative impact on the midwife–woman relationship because male partners speaking on behalf of women was viewed as an act of male dominance and control that could create more barriers to establishing a relationship and getting to know the women [45].

### 3.3. Ensuring Normality and the Bond between Baby and Parents While Protecting Life

#### 3.3.1. Taking Care through Measurements and Tests

Measuring and testing the woman and baby helps to assure the woman that everything is normal and being taken care of by the midwife. Naturally, the main outcome measure used by women and midwives was the successful birth of a live, healthy baby [48], but the approach of midwives during ANC varied, with some being more interested in gestational age and pregnancy than the person. Professionals highlighted the importance of following guidelines for screening in pregnancy to ensure better outcomes for women and babies [4,5,48,50] and a belief that physiologic birth is a natural process and that women’s bodies are designed for childbirth [48,50].

Gestation-related conversations with women were short in duration and narrowly focused but some midwives concentrated on this aspect routinely as the guide for care (examples are blood pressure, auscultation of the woman’s stage of pregnancy and the fetal heart beat [4]. They helped women understand the situation by giving them information on normal blood pressure and weight and fetal heart zone rates [6].

There were challenges, too. Too little information was given to women, and a routine hospital ultrasound scan was described as “getting a picture of the baby” [46] without the real purpose of the scan (risk screening) being explained. Different kinds of measurements showed the midwives’ professional skills but also how they dominated the conversation by having the women in a semi-recumbent position as the only opportunity for further conversation with them and other family members [4]. Some of the midwives also underestimated the importance to women of explanations about the purpose of antenatal tests or screening procedures or advice about perinatal care of stitches or bruising or breastfeeding [48].

#### 3.3.2. Promoting the Bond with the Unborn Child by Offering Information and Guidance

Midwives promoted awareness of the child and its wellbeing in the woman’s womb and the development of the mother’s relationship with the unborn child [17,20]. Midwives helped women observe and get to know the baby: “… it is important to talk about the child in the womb… what it looks like… and the dreams about it… to recognize the child.” [20]. As in the study by [17], they guided women suggesting, for example, that it was important for them to lie quietly in bed at the end of the day with their hands on their bellies to become used to the baby and its movements. They also promoted the future psychological wellbeing of the child by encouraging women to talk about and reflect on parenthood [17,20]. One aim of parenting education was to foster the parents’ relationship with the unborn child and strengthen the parent’s capacity to handle the process of childbirth [20].

#### 3.3.3. Cultural Challenges and Preventing Possible Health Risks

Even though midwives faced challenges in their work caused by different cultural norms, they tried to prevent health risks for the child, while supporting parenthood and trying to understand people from different backgrounds. This requires more knowledge and training. Andersson et al. [42] found a need for more individual work to prepare parents from different cultures for parenthood [42]. Challenges were presented by the different parenting roles of fathers and family dynamics, the difficulty of creating a trustful professional relationship and getting to know women despite interference from their domestic partners [45]. It was hard to provide close personal care when interpreters were present because it impacted on the relationship between the women and midwives and the exchange of information [7].

Despite the priority given to the women’s views by midwives, women seemed unware that their partner’s involvement in their ANC might affect their relationship with midwives and instead framed the partner’s involvement as positive and caring. With male-led interaction, it was difficult to get first-hand information from women even though they found it a normal way to communicate. “Woman seems a little nervous… looking to partner for answers when midwife asks questions (despite good English fluency). Partner answers most of the questions for woman. Midwife tries to engage woman by directing questions at her—woman turns to partner and waits for him to answer….” [45].

Sometimes the pregnancy and childcare advice given by a family member, such as a mother-in law, was perceived as a potential barrier to the midwife–woman relationship as described in the study by Goodwin et al.: “… all they’re hearing is what the mother-in-law or the family tell them… and they’re taking that as gospel. And you’ve got a real battle to say “just because grandma said it doesn’t mean to say it’s right!” There were also some practices that impacted negatively on the midwife-woman relationship, such as dressing newborns with glass/string bracelets, fasting whilst pregnant, shaving the newborn’s head or placing honey on the newborn’s tongue immediately after birth [45]. Other challenges included offering group-based antenatal care, which was objected to by some immigrants, language barriers, well-educated parents and parents with certain problems [42], but also women from a different culture (Pakistani) not being willing to follow the midwife’s advice at home [45]. Despite the challenges, for some healthcare providers it was important to offer services in a culturally sensitive manner [5].

### 3.4. Promoting the Health and Wellbeing of the Family Today and in the Future

#### 3.4.1. Providing and Sharing Information and Support through Parenting Education

Midwives help parents focus on their new life situation by providing them with information and skills through parenting education, involving understanding antenatal care [6], developing the relationship with the unborn child [20], preparing for parenthood and childbirth [20,50], including gender perspectives and sexuality [20,50], being an adult in a changed relationship [49], information and advice on labor, pain relief, breastfeeding [20] and taking care of the child [49].

One important thing regarding parenting education mentioned by midwives and obstetricians was that such education is health education as well as health promotion: “... we have to think about prevention… our responsibility is to be health educational… a public-health approach” [20]. Parenting education also involves midwives supporting parents’ individual time for transition, strengthening their confidence and reducing stress. Maternity care providers felt a shared responsibility to educate women during pregnancy (exercise and healthy lifestyle preparation, emotional and physical preparation, childbirth preparation and comfort and coping measures in labor) [50].

Midwives also used new ways to work with parents, such as group-based ANC [43]. They were worried about the privacy of parents using digital communication and social media [5], but also about information provided online. They did not inform women about evidence-based pregnancy-related websites [43].

#### 3.4.2. Seeing the Importance of Family Members and Networks and Accepting Them in Antenatal Care

Some of the midwives found it important that the mother got as much support as possible from the father/partner but also from other family members and encouraged them to come to ANC meetings with the woman. Making them feel welcome included attending to the immediate comfort needs of accompanying family members and children [4]. Saftner et al. [50] found it was important to also include friends in discussions because social support networks play a part in individualizing care and improving confidence and the positive encouragement that women receive.

While family members were a source of support for women, some midwives perceived this involvement as having a negative impact on the midwife–woman relationship [45]. Difficulties arose because of different cultures, such as those marked by male dominance and speaking on behalf of women [45]. In Australian aboriginal communities, if an expectant or new mother has her own mother present, it could displace the father of the baby because it is difficult for him to be involved at the same time [49]. The pregnancy and childcare advice given by a family member such as a mother-in law was perceived as a barrier to the midwife-woman relationship and competition on right or wrong information [45].

#### 3.4.3. Helping Parents Connect with Other People and Get Peer Support

Although midwives find it important to respect privacy, they also see the importance of families extending their social contacts and having family members around them and social networks. The parenting education provided in ANC helps to create networks that are a source of support and peer support [20,42], and in a group parents can extended knowledge and understanding with different parents questions and thoughts [42].

Many mothers-to-be live far from their relatives and their stay in the postnatal ward is brief. The knowledge they need can be communicated through networks which are formed during parenting education [20]. The midwives understand the benefits for parents of group-based antenatal care are getting to know other parents and the opportunity for peer support, but some of the midwives valued more individual meeting because “many meetings take place in groups today; we should protect the individual encounter” [42]. Fathers were also encouraged to participate in services and support programs in new parent groups [49]. For private discussions, midwives also offer the possibility for additional office visits in conjunction with group-based ANC [6]. Today, social and multi-directional media can also be used and offer the benefits of peer exchange and community participation and support [12].

#### 3.4.4. A Metaphor

A metaphor is a tree in its entirety, with its branches and deep roots. The trunk embodies key components of nursing: a holistic conception of human beings, ethical principles, the theoretical background, governing principles and guidelines. Deep in the roots, they form the core and foundation of the care provided. The trunk of the almost leafless tree could represent the situation at the outset of antenatal care, when cooperation with the woman, father/partner and family begins to be built. On the branches there are gradually opening buds and leaves (awakening). The shoots could illustrate seeing parents as individuals, being interested, listening, communication, building and supporting confidence, encouraging participation, promoting self-determination and decision-making, solving problems and supporting coping.

The buds opening out into leaves represent progress on the antenatal care journey, such as encountering new things, caring, guiding, and monitoring health and wellbeing ideas. They illustrate measuring, testing, being professional in knowing and following the guidelines, promoting and ensuring better outcomes for mothers and babies, focusing on the mother and pregnancy, fetus and family. The involvement and participation of the family varies according to the size of the buds and leaves. The open leaves also illustrate ensuring that the antenatal care pathway goes in the right direction in providing support. All stages involve promoting health and wellbeing.

Falling leaves could illustrate the support provided by social welfare and healthcare professionals relating to changes in parents’ lives and the opportunity to see things in a new way and help turn them in a better direction, oriented firmly to the future. The pathway can be rebuilt with new buds. Falling leaves can also represent the various challenges encountered by professionals, requiring new buds for solutions and sensitivity to change. They can also illustrate inadequate antenatal care and the lack of it. Can we meet the need with new buds?

The seasons illustrate the cycle of a person’s life in different situations. With antenatal care, new buds open as the life of a family with a baby enters a new cycle, which is partly based on the previous one. The overarching theme is expressed as “helping the woman and her partner prepare for their new life with the child by providing individualized, shared care, firmly grounded and with a view of the future”.

## 4. Discussion

This meta-ethnography illuminates caring and the relationship with the woman, partner and family in antenatal care (ANC) from the perspective of midwives and nurses. The chosen studies described the experiences of midwives and nurses working in ANC, interacting with and caring for their clients, primarily women but also fathers/partners, sometimes in online and cultural contexts, providing parenting education and using various models such as the connected-care model. Caring in ANC is a multidimensional process for midwives and nurses, who have to deal with many different demands concurrently, which can result in ANC being fragmented. A high level of professional ethics should always be part of ANC [2].

In order to promote the health of the whole family, it would be valuable for the midwife and nurse to make time to listen to their views on their health and factors related to parenthood. The current research shows that the relationship between women and professionals is vital for women to engage in their ANC, but the active involvement of fathers/partners is also considered important. The results reveal that ANC begins with midwives and nurses having a conscious plan when first meeting pregnant women, recognizing the importance of creating a trustful and confidential relationship. Caring entails gaining the trust of each woman early on as the basis for future visits to the maternity clinic: treating the woman as a human being, without emphasizing professional authority, making her feel welcomed and that the midwife or nurse is interested in her and is there to provide help and support. The relationship was built carefully by creating a positive atmosphere, concentrating on the individual and gaining the woman’s commitment to regular visits to the clinic [4,17,44,48,49,50]. This safeguards the wellbeing of the developing baby and the mother during pregnancy but also facilitates the exchange of necessary information in preparing for parenthood and the giving of support for the future.

It was important for midwives to connect and create a relationship with both parents and actively get to know the father/partner, too. This involves midwives taking note of men’s receptiveness to ANC since engaging fathers is part of the midwife’s role [49]. Some studies present care models facilitating development of rapport with the family by enabling more contact, engagement and support for fathers [4,5]. However, some of the midwives did not even mention the importance of involving women’s partners [48], and fathers were outsiders in various ways [5,10,11,12,13,43,45,47,48]. Yet the fathers themselves are interested in positive fathering and see themselves as much more than just passive supporters of their partners and want to be genuinely engaged [15]. According to [4], the rituals and practices, together with knowledge of interpersonal skills, are integral to effective women-centered care. Many things connected with ANC are common to the expectant couple/parents and the whole family. Even though midwives and other antenatal staff use a range of strategies in their services, their attempts to tailor health information to individual needs are frequently based on incomplete information about patients’ health literacy, may be inconsistent in delivery and content and are seldom assessed to determine whether communication has been understood or led to a change in patient behavior [31].

While emphasizing women-centered care is important, the question also arises of whether fathers may be left in the background as a result. There may be concern as to whether such an approach leads professionals to see caring as only women-centered rather than parent- and family-centered. Antenatal care meetings (with the mother, father/partner) are an important part of midwives’ professional reflection and competence both from a women-centered and family-centered point of view, despite the emphasis being on women-centered care. Ultimately, however, it is a question of meeting and engaging with people. It is important to remember that International Confederation of Midwives (2014) the code addresses the midwife’s ethical mandates in keeping with the mission, the international definition of the midwife, and standards of ICM to promote the health and wellbeing of women and newborns within their families and communities. The father’s/partner’s importance in providing support for the pregnant woman is central but growing into fatherhood is also mentally and emotionally demanding. Parenting education was of great significance in helping parents prepare together for a lifelong commitment and giving them the opportunity to reflect and talk about parenthood during the pregnancy [20]. Providing ANC involves dealing with diverse families, and there are also challenges related to resources and the conditions in which ANC is carried out. The extent to which fathers are invited and encouraged to take part in ANC with the woman varies. Given the key importance of fathers, visiting the maternity clinic enables them to grow into parenthood alone and together with the woman, helps them create a relationship with the unborn child and improves their readiness to care for the child and support their spouse. While [34] found that fatherhood was not discussed as much as motherhood during ANC, the current research revealed that some of the midwives and nurses considered it important to connect with the fathers, too, despite the difficulties involved. It is important to openly discuss how to solve challenges and support good parenting for the benefit of the family. In the case of suspected or actual violence, it is essential to quickly provide special support and professional counselling.

Midwives met cultural challenges and prevented possible health risks. Midwives working in a cultural context encountered challenges related to tailoring care to individual needs, overcoming communication barriers and enabling partner involvement, dealing with stereotypes and also addressing varied levels of health literacy [27]. Culture-related challenges affecting the relationship and communication between midwife and parents are common in ANC [28,50]. Wikberg et al. [26] found that female nurses were seen as professional friends, and the conflicts encountered were resolved, which in turn promoted caring. It is evident that midwives and nurses need more training ⌈7,46,50⌉ and also that a better understanding of different cultures and habits ⌈7,46⌉ and traditional practices can promote caring in ANC by midwives [45].

Checks and measurements done in connection with monitoring the baby and pregnancy are key aspects of ANC, but the focus and goals of care are wider. Midwives promoted awareness of the child and its wellbeing in the woman’s womb and the development of the mother’s relationship with the unborn child. As part of the process of growing into parenthood, the bond with the unborn child could also be promoted by guiding parents to respond to the baby’s movements by speaking, using music and preparing for the arrival of the baby by making space for it at home, etc. Ultrasound examination is much more than getting a picture of the baby and/or the results [46].

There was no mention of a salutogenesis approach, as such, in support of the expectant parents’ sense of coherence as a means of promoting the whole family’s health and wellbeing. But there were many different views on comprehensibility, manageability and meaningfulness—for example, regarding sharing information, providing information on emotional experiences and expectations of pregnancy, birth and parenthood, and the relationship between the couple and the expectant parents’ own parents [34]. Midwives supported women’s self-determination [4], encouraging problem-solving and coping [17,44] and negotiating decision-making about care choices [44,46]. A birth plan has been found very important for discussion of past personal experiences, labor and birth to help women orient themselves with confidence to the new situation [50], even though it was not always made available later [53]. There is a great need for women to have the opportunity to discuss their birth plan with the midwife and their need for support and information. Vinje et al. [38] recommend that education should include salutogenesis as a body of knowledge, as a continuous learning process, as a way of working, and as a way of being. Equal emphasis on what to do, how to do it, how to be it is a key factor in succeeding in training health professionals in salutogenesis [38]. Midwives and nurses can use a salutogenic approach during ANC to support the parents’ sense of coherence: comprehensibility (cognitive), manageability (behavioral) and meaningfulness (motivational) for the movement towards the whole family’s health and wellbeing. Comprehensibility can help the parents to better understand the transition and the new situation as a parent-to-be, being an active partner during care and strengthening the relationship with the child and each other. It means giving information, caring and supporting parents’ own wisdom as a way of problem-solving and coping. Manageability can help parents to be more involved in their life situation and the changes, also in the future, to support their own decision-making and management, and the health and wellbeing of the whole family. This could be supported in ANC by parenting education provided in various ways, such as groups and online. Meaningfulness can lead to a lifelong commitment to taking active care of themselves, providing a strong base for the health and wellbeing and health literacy of the family. If parents-to-be have a strong sense of coherence, they want and are motivated to cope as a parent and family, believe that their challenges are understood and that the resources to cope are available also in their future family life.

Mothers may have a different view of holistic care than nurses. Despite the fact that midwives tried to help women take part in their own antenatal care in various ways to support women’s self-determination, many younger women preferred to defer to the professionals rather than be involved actively in care decisions as they were not used to making choices [48]. It would be very beneficial to engage pregnant women in their own care and decision-making. Encouraging women to make their own decisions helps to enhance their self-confidence while reducing the emphasis on professional authority. After the birth of the child, parents have to face and make independent decisions that require self-confidence. The meta-synthesis brought out language-related problems, not only because of different languages but also in communication or choice of words. Understandable information and instructions are essential in carrying out care. Some of the nurses were more sensitive in their use of language and made a conscious effort to take it into consideration when meeting clients. It has previously been shown that language- and communication-related problems may cause a lack of trust, with a negative impact on caring [26].

Parenting education is important for parents in strengthening their readiness for parenthood, enabling them to ask questions and talk about their concerns, and providing them with information and skills regarding, e.g., caring for the child, health-promoting choices, and the couple’s relationship. It was considered particularly important to meet others in the same life situation and to receive peer support. In the best cases, these relationships may develop into lifelong friendships. There is also an evident need to provide more training for midwives and nurses [7,45,49] that better understanding of traditional practices can promote caring in ANC [45]. Nurses are worried about the impact of digitalization on maternity care in relation to verbal and non-verbal communication [17,43], lost connectivity and control [43], and the privacy aspect [5,43], but also about not having enough skills, thus wanting more education and training [5,42,43,49]. It is time to consider providing more individual ANC services together with traditional appointments. There is a great need to use digital solutions as a way of connecting with parents and offering flexible services. This need has grown as a result of the COVID-19 pandemic. Is it time for midwives and nurses to move towards a salutogenic approach and away from midwife-dominated to midwifery-led antenatal care? [54] Is it also time to provide midwives and nurses with enough support and resources to turn fragmented ANC into holistic and salutogenic ANC? This is a way of strengthening antenatal care towards salutogenic approach.

## 5. Strengths and Limitations

The strengths of this meta-ethnography lie in the targeted, systematic and comprehensive search process, involving trying different search words and finally using them as recommended by professionals at the university library. The data was carefully chosen based on a three-level meta-ethnography: research participants’ (social and health care professionals’) experiences, the researcher interprets these experiences, and the meta-ethnographer reinterprets the researcher’s concepts [32]. We also had clear inclusion and exclusion criteria for the studies, used quality appraisal [55] and reporting taking account of the eMERGe guidelines [40]. The confidence in findings assessed from qualitative evidence syntheses by GRADE-CERQual [39,53] (Table 6).

Our international team had a leader with a lot of experience of this kind of research and expertise in mentoring with a background in public health nursing. The other members had backgrounds in midwifery, public health nursing and nursing. Since social and health care professionals in different countries work in ANC, all the authors knew the context of this research. They had also worked in ANC in this nursing context and taught students and professionals in maternity and family care. As a working research team, we collaborated effectively with enough time to stay close to the data and avoid our own interpretations, which might have been a problem if the process had been hurried. But at the same time, having enough time together for discussion was complicated because of many other commitments. Author is aware of more resent research on this topic but decided to focus on this carefully selected and analyzed date. There would be various ways of reporting and describing the findings. In the current research, the findings are presented as text with some quotations and descriptions [32,33].

## 6. Conclusions

Caring in antenatal care (ANC) as provided by midwives/nurses is being totally present, listening and using multidimensional professional competence but also being open--minded to new aspects and knowledge. Creating a positive, confidential and open relationship with the woman and man/partner and the whole family is fundamental to ANC.

The health promotion and positive health aspects should be considered an important part of supporting parents and the whole family now and in the future. A more conscious salutogenic approach to ANC would lead to more favorable results. This could be a fruitful research topic in the future.

There is a need to provide midwives/nurses with enough time to allow them to concentrate on specific needs of different kinds of families in ANC and to be as supportive as possible during the appointments and classes but also training for midwives to make them more familiar with online and other options.

## Figures and Tables

**Table 1:** The eMERGe meta-ethnography reporting guidance [40,41].

Criteria Headings	Reporting Criteria	Pages
**Phase 1 Selecting meta-ethnography and getting started** *Introduction* 1. Rationale and context for the meta-ethnography 2. Aim(s) of the meta-ethnography 3. Focus of the meta-ethnography 4. Rationale for using meta-ethnography	Describe the gap in research or knowledge to be filled by the meta-ethnography and the wider context of the meta-ethnography Describe the meta-ethnography aim(s) Describe the meta-ethnography review question(s) (or objectives) Explain why meta-ethnography was considered the most appropriate qualitative synthesis methodology	1–3
**Phase 2 Deciding what is relevant** *Methods* 5. Search strategy 6. Search processes 7. Selecting primary studies 8. Outcome of study selection	Describe the rationale for the literature search strategy Describe how the literature searching was carried out and by whom Describe the process of study screening and selection, and who was involved Describe the results of study searches and screening	5–7, 9–14
**Phase 3 Reading included studies** *Methods* 9. Reading and data-extraction approach *Findings* 10. Presenting characteristics of included studies	Describe the reading and data-extraction method and processes Describe characteristics of the included studies	5–8, 9–14
**Phase 4 Determining how studies are related** *Methods* 11. Process for determining how studies are related *Findings* 12. Outcome of relating studies	Describe the methods and processes for determining how the included studies are related: -Which aspects of studies were compared -How the studies were compared Describe how studies relate to each other	8
**Phase 5 Translating studies into one another** *Methods* 13. Process of translating studies *Findings* 14. Outcome of translation	Describe the methods of translation: -Describe steps taken to preserve the context and meaning of the relationships between concepts within and across studies -Describe how the reciprocal and refutational translations were conducted -Describe how potential alternative interpretations or explanations were considered in the translations -Describe the interpretive findings of the translation	8, 15–23
**Phase 6 Synthesizing translations** *Methods* 15. Synthesis process *Findings* 16. Outcome of synthesis process	Describe the methods used to develop overarching concepts (‘synthesized translations’), and describe how potential alternative interpretations or explanations were considered in the synthesis Describe the new theory, conceptual framework, model, configuration, or interpretation of data developed from the synthesis	7–8, 15, 23
**Phase 7 Expressing the synthesis** *Discussion* 17. Summary of findings 18. Strengths, limitations and reflexivity 19. Recommendations and conclusions	Summarize the main interpretive findings of the translation and synthesis, and compare them to existing literature Reflect on and describe the strengths and limitations of the synthesis: Methodological aspects: for example, describe how the synthesis findings were influenced by the nature of the included studies and how the meta-ethnography was conducted Reflexivity—for example, the impact of the research team on the synthesis findings Describe the implications of the synthesis	15–23, 15, 26, 28

**Table 2:** Key terms for search.

The First	The Second	The Third
(Prenatal care and Visit*) or (antenatal care and visit*) and Midwi*	(Prenatal care and Visit*) or (antenatal care and visit*) and Public Health Nurs*	Prenatal care and Visit* or visit and Midwiv* or Public Health Nurs*and qualitative

**Table 3:** Quality assessment of selected studies (*n* = 16). (Abbreviations: Y = yes; N = no; - = cannot tell).

Authors	Clear Aims	Appropriate Methodology	Appropriate Design	Appropriate Recruitment Strategy	Appropriate Data Collection	Adequate Consideration on Relationship between Researcher and Participants	Ethical Considerations	Rigorous Data Analysis	Clear Statement of Findings	The Value of the Research
Alden et al. (2008) [20]	YY	YY	YY	YY	YY	Y-	YY	YY	YY	YY
Andersson et al. (2014) [42]	YY	YY	YY	YY	YY	Y-	YY	YY	YY	YY
Aquino et al. (2015) [7]	YY	YY	YY	YY	YY	Y-	YY	Y-	YY	YY
Baron et al. (2018) [6]	YY	YY	YY	YY	YY	--	YY	YY	YY	YY
Browne et al. (2014) [17]	YY	YY	YY	YY	YY	--	YY	YY	YY	YY
Dalton et al. (2014) [43]	YY	YY	YY	YY	YY	--	Y-	Y-	YY	YY
Dove et al. (2014) [44]	YY	YY	YY	YY	YY	Y-	YY	YY	YY	YY
Goodwin et al. (2018) [46]	YY	YY	YY	YY	YY	--	YY	YY	YY	YY
McCourt (2006) [46]	YY	YY	YY	YY	YY	--	--	YY	YY	YY
Olsson et al. (1996) [47]	YY	YY	YY	YY	YY	Y-	Y-	YY	YY	YY
Proctor (1998) [48]	YY	YY	YY	YY	YY	--	-Y	yy	yy	YY
Rominov et al. (2017) [49]	YY	YY	YY	YY	YY	YY	YY	YY	YY	YY
Saftner et al. (2017) [50]	YY	YY	YY	YY	YY	--	YY	YY	YY	YY
Sword et al. (2012) [5]	YY	YY	YY	YY	YY	YY	YY	YY	YY	YY
Withford et al. (2014) [53]	YY	YY	YY	YY	YY	--	YY	YY	YY	YY
Wright et al. (2018) [4]	YY	YY	YY	YY	YY	-Y	YY	-Y	YY	YY

**Table 4:** Characteristics of selected studies (*n* = 16) and key findings.

Author(s), Year, Country	Study Design and Aim(s) of Study	Sample of Participants	Context	Method of Data Collection and Analyses	Key Findings
Ahlden et al. (2008) [20] Sweden	Phenomenograpy To describe perceptions of parenthood education (PEC) among midwives and obstetricians in charge of antenatal care in Sweden.	*n* = 25 Midwives (*n* = 13) and obstetricians (*n* = 12)	Swedish antenatal care	Focus group interviews Thematic analysis	There is a strong belief in PEC and the overall aim was considered to be support in the transition to parenthood. A good transition is influenced by several factors such as expectations, levels of knowledge, and the parents’ environment. Father-to-be sessions with male leaders is very important.
Andersson et al. (2014) [21] Sweden	An interview study To investigate and describe antenatal midwives’ perceptions and experiences of their current work, with a special focus on their opinions about GBAC (group-based antenatal care).	Midwives (*n* = 56)	52 antenatal clinics.	Triangulation Descriptive statistics Content analysis	The midwives were satisfied with their work in antenatal care but have reservations concerning lack of time and content, individual care and quality of parental classes. They had strong opinions about women’s suitability for the model. GBAC can be more discussion-based and adapted to modern parents.
Aquino et al. (2015) [7] UK	Qualitative research To explore a cohort of midwives’ experience of providing care for BME (Black and minority) women, focusing on their views on the relationship between maternal health inequalities and service delivery.	Midwives (*n* = 20)	Hospital	Semi-structured interviews Thematic analysis	Many minority women’s complex care needs were identified during pregnancy by midwives. Whilst midwives strove to provide high-quality, individualized care for all women by being sensitive and responsive to women’s individual needs, many barriers were present such as organizational, language and cultural differences.
Baron et al. (2018) [6] USA	Qualitative evaluation To explore the perspectives of patients, RNs (Registered Nurse), and other providers regarding a new prenatal connected care model for low-risk patients aimed at reducing in-office visits and creating virtual patient-RN connectors.	Patients (*n* = 41) Other providers (*n* = 17), (physicians *n* = 8 and certified nurse midwives (CNMs) *n* = 9)	10 units/hospital	Semi-structured interviews Thematic analysis	By reducing the number of scheduled in-office visits and increasing the RN’s role in patient management and education, the new parental care model sought to make more efficient use of the health care team. It also provides patients with greater flexibility and control of their care. The new model valued connectedness and the relationship with the connected care RNs. The connected care RNs appreciated being able to work to a fuller scope of practice.
Browne et al. (2014) [17] Australia	Qualitative To explore midwives’ communication techniques intended to promote a wellness focus in the antenatal period, this study identifies strategies midwives use to amplify women’s own resources and capacity, with the aim of reducing antenatal anxiety.	Midwives (*n* = 14)	Multiple hospitals and community settings	Focus group interviews Means of generating data	The midwives want to make the system of ANC (antenatal care) work for women. Wellness-focused care is both a responsibility and a right. The midwives used individually a variety of strategies in ANC specifically intended to facilitate women’s capabilities, to employ worry usefully and to reduce anxiety.
Dalton et al. (2014) [43] Australia	Triangulation To investigate midwives’ attitude and experiences of ICT (information and communication technologies) use to identify potential causal factors that limit usage.	Midwives (*n* = 40)	Single hospital	Focus group interviews. Thematic and statistical analyses.	The midwives recognize both potential benefits and possible risks in the use of ICT. The problems were lack of training, the perceived legal risks associated with social media, potential violations of patients’ privacy, misdiagnosis and misunderstanding between midwife and client.
Dove and Muir-Cocrane (2014) [44] Australia	Ethnography To examine how midwives and women within a continuity of care midwifery program in Australia conceptualized childbirth risk and the influences of these conceptualizations on women’s choices and midwives’ practice.	Midwives (*n* = 8), an obstetrician (*n* = 1) and women (*n* = 17)	Antenatal appointments	Semi-structured interviews, observation.	The relationship between the midwives and the women for the success of the continuity of care is important. Identities as safe mothers and practitioners developed out of intersubjective processes within their relationship.
Goodwin et al. (2018) [45] UK	Ethnography To address the paucity of literature examining the midwife-woman relationship for migrant women by exploring the relationship between first-generation migrant women and midwives focusing on identifying the factors contributing to these relationships, and the ways in which these relationships might affect women’s experiences of care.	Midwives (*n* = 11) and Pakistani migrant women (*n* = 9) in early pregnancy.	Community-based antenatal clinics	Interviews, Observation Thematic analysis	The midwife-woman relationship was important for maternity care, pregnancy outcomes and staff satisfaction. The midwives allowed the partner to be present, but some women seem to be unaware of their partner’s involvement. Numerous social and ecological factors influenced this relationship, including the family relationship, culture and religion, differing health-care systems, authoritative knowledge and communication of information.
McCourt (2006) [46] UK	An observational approach To explore whether, or how, the recent reforms to promote more women-centered care would have a positive impact on interaction between midwives and their clients, particularly regarding the areas of information and choice which have been so extensively critiqued.	Booking visits with pregnant women (*n* = 40) and midwives (*n* = 40)	Hospital clinic, GP clinic and women’s homes.	Observations Structured and qualitative analysis	The interactional patterns differed according to the model of care, i.e., conventional or caseload, and setting of care. A continuum of styles of communication was identified, with the prevalent styles also differing according to location and organization of care. The caseload visits showed less hierarchical and more conversational form and supported women’s reports and gave them greater information, choice and control.
Olsson et al. (1996) [47] Sweden	Qualitative To describe antenatal “booking” interviews as regards and illuminating the meaning of the ways midwives and expectant parents relate to each other.	Midwives (*n* = 5) Women (*n* = 5) Fathers (*n* = 2)	Midwifery clinics at five health centers in Sweden.	Video-recorded antenatal booking interviews. Content analysis Phenomenological hermeneutic analysis	There are two views on providing ANC. The former focused on the physical process of pregnancy and birth; the latter on the process of becoming parents including the psychological and social circumstances in addition to the physical. The ways midwives related were considering and disregarding the uniqueness of the expectant parents. The expectant fathers seemed like strange visitors in the women’s world.
Proctor (1998) [48] UK	Qualitative To identify and compare the perceptions of women and midwives concerning women’s beliefs about what constitutes quality in maternity services.	Midwives (*n* = 47) Women (*n* = 33)	Maternity clinics Hospital and community based	Focus group interviews Thematic analysis	The ANC was characterized primarily by a need for information, understanding and reassurance. Understanding and respect between women and midwives are important aspects of maternity care. It is important to know midwife before labor. The midwives overestimated the importance women attached to discussing information leaflets during pregnancy.
Rominov et al. (2017) [49] Australia	Multi-method approach To describe midwives’ perceptions and experiences of engaging fathers in perinatal services.	Midwives (*n* = 106)	Perinatal services and fathers	Semi-structured telephone interviews Descriptive analyses	Engaging fathers is part of the midwives’ role and they acknowledged the importance of receiving education to develop knowledge and skills regarding fathers. Being father-inclusive should be given more emphasis by midwives. The midwife-led continuity of care as a model could be of benefit to fathers.
Saftner et al. (2017) [50] USA	Qualitative descriptive study Grounded theory To explore MCPs’ (Maternity Care Providers) beliefs and attitudes about physiologic birth and to identify components of antenatal care that providers believe may impact on a woman’s confidence in physiological labor and birth.	Maternity care providers (*n* = 31) Certified Nurse-Midwife (CNM) *n* = 14 Family medicine doctors *n* = 8 Obstetrician- Gynecologist *n* = 9	Maternity care	Semi-structured interviews Inductive coding	Maternity care providers support a physiological approach to labor and birth and wish to enhance outcomes for mothers and babies. They would like to provide more information to women about the care during birth and support for women’s choices.
Sword et al. (2012) [5] Canada	Qualitative descriptive approach To explore women’s and care providers’ perspectives of quality prenatal care to inform the development of items for a new instrument, the Quality of Prenatal Care Questionnaire.	Prenatal care providers (*n* = 40), including obstetricians, family physicians, midwives and nurses (practiced in obstetrics/maternity care minimum 2 years). Pregnant women (*n* = 40).	Five urban centers across Canada	Semi-structured interviews Inductive approach	Interpersonalized care is an approachable interaction style that involves taking time. Having a meaningful relationship with prenatal care may be fundamental to the quality of care and involves trust. The appointment flexibility and clinical knowledge of professional belongs in the provision of quality care.
Withford et al. (2014) [51] UK	Exploratory qualitative and longitudinal study To consider use of a standard birth plan section within a national, woman-held maternity record.	Women (*n* = 42) Maternity service staff (*n* = 24) Midwives *n* = 15 (hospital *n* = 6) and community and/or midwife-led unit *n* = 9) Obstetricans *n* = 6 Grade *n* = 4 (Consultant *n* = 4, specialist trainee obstetrician or below *n* = 2) General practitioner *n* = 3	Antenatal clinics	Interviews Thematic analysis	The staff and women were generally positive about the provision of the birth plan with the record. The birth plan could stimulate discussion about labor and birth options, and support communication about women’s preferences and concerns. It could also serve to facilitate and enhance women’s awareness of staff responsiveness to women during pregnancy and labor.
Wright et al. (2018) [4] Australia	Contemporary focused ethnography To address the question: “How are the principles of woman-centered care applied in the hospital antenatal care setting?”	Midwives (*n* = 16)	Six different public antenatal clinics and antenatal consultations.	Interviews Observation Thematic analysis	Behaviors that promote time for women to express their feelings and needs, particularly during ANC, are important and are key to supporting the woman’s self-determination. To assist midwives in providing woman-centered conversations and care, managerial support may also be required with realistic timeframes and expectations.

**Table 5:** Themes developed during the meta-ethnography to integrate findings.

The Overarching Theme: Helping the Woman and Her Partner Prepare for Their New Life with the Child by Providing Individualized, Shared Care, Firmly Grounded and with a View of the Future				
Core Themes	Supporting the Parents to Awaken to Parenthood and Creating a Firm Foundation for Early Parenting and Their New Life Situation	Guiding Parents on the Path to Parenthood and New Responsibility	Ensuring Normality and the Bond between Baby and Parents While Protecting Life	Promoting the Health and Wellbeing of the Family Today and in the Future
Themes	*Welcoming, being actively present and available* *Connecting and creating a relationship with both parents* *Building continuity and trust in the relationship between midwife and women* *Actively getting to know the father/partner to encourage and engage them* *Recognizing the importance of language in providing support and dealing with challenges*	*Listening to each person to encourage participation* *Supporting women’s self-determination* *Building and supporting the confidence of fathers/partners*	*Taking care through measurements and tests* *Promoting the bond with the unborn child by offering information and guidance* *Cultural challenges and preventing possible health risks*	*Providing and sharing information and support through parenting education* *Seeing the importance of family members and networks and accepting them in antenatal care* *Helping parents connect with other people and get peer support*
Authors	Olsson et al. [34] Ahlden et al. [20] Sword et al. [5] Andersson et al. [42] Dove et al. [44] Browne et al. [17] Rominov [49] Saftner et al. [50] Goodwin et al. [45] Wright et al. [4]	Olsson et al. [47] Proctor [48] McCourt [46] Sword et al. [5] Rominov et al. [49] Saftner et al. [50] Andersson et al. [42] Browne et al. [17] Dove et al. [44] Withford et al. [51] Aquino et al. [7] Saftner et al. [50] Goodwin et al. [45] Wright et al. [4]	Proctor [48] McCourt [46] Sword et al. [5] Andersson et al. [42] Aquino et al. [7] Saftner et al. [50] Goodwin et al. [45] Wright et al. [4]	Ahlden et al. [20] Sword et al. [5] Dalton et al. [43] Andersson et al. [42] Rominow et al. [49] Saftner et al. [50] Baron et al. [6] Goodwin et al. [45] Wright et al. [4]

**Table 6:** CERQual Qualitative Evidence Profile.

The Finding of the Review		Studies Contributing to the Review Finding	Assessment of Methodological Limitations	Assessment of Relevance	Assessment of Coherence	Assessment of Adequacy	Overall CERQual Assessment of Confidence and Summary
Supporting the parents to awaken to parenthood and creating a firm foundation for early parenting and the new life situation	***Welcoming**, being actively present and available* ***Connecting** and creating a relationship with both parents* ***Building** continuity and trust in the relationship between midwife and women* ***Actively** getting to know the father/partner to encourage and engage them* ***Recognizing** the importance of language in providing support and dealing with challenges*	Olsson et al. [34] Ahlden et al. [20] Sword et al. [5] Andersson et al. [42] Dove et al. [44] Browne et al. [17] Rominov [49] Saftner et al. [50] Goodwin et al. [45] Wright et al. [4]	Minor methodological limitations. Minor concerns on relationship between researcher and participants, minor concerns about analysis and ethical considerations and clarity about reflexivity.	Minor concerns about relevance. The primary studies support a review finding is applicable to the context. The ANC by midwives/nurses in different places/context and different countries.	Minor concerns regarding coherence. Data reasonably consistent within and across studies. The data from the primary studies (carefully chosen) and a review finding synthesises the data good.	Minor concerns about adequacy of data. The participant and the data are presented. The whole data is rich with 16 studies and it described though some quotations.	Moderate confidence. The review finding is a reasonable representation of the phenomenon of interest. The minor methodological considerations, minor concerns about relevance, coherence and adequacy of data.
Guiding parents on the path to parenthood and new responsibility	***Listening** to each person to encourage participation* ***Supporting** women’s self-determination* ***Building** and supporting the confidence of fathers/partners*	Olsson et al. [47] Proctor [48] McCourt [46] Sword et al. [5] Rominov et al. [49] Saftner et al. [50] Andersson et al. [42] Browne et al. [17] Dove et al. [44] Withford et al. [51] Aquino et al. [7] Saftner et al. [50] Goodwin et al. [45] Wright et al. [4]	Minor methodological limitations. Minor concerns about data analysis, clarity about reflexive, a few ethical clarities and relationship between researcher and participants.	Minor concerns about relevance. The primary studies support a review finding is applicable to the context. The ANC by midwives/nurses in different places/context and different countries.	Minor concerns regarding coherence. Data reasonably consistent within and across studies. The data from the primary studies (carefully chosen) and a review finding synthesises the data good.	Moderate concerns about adequacy of data. The participant and the data are presented. The whole data is rich with 16 studies and it described though some quotations.	Moderate confidence. The review finding is a reasonable representation of the phenomenon of interest. The minor methodological considerations, minor concerns about relevance, coherence and adequacy of data.
Ensuring normality and the bond between baby and parents while protecting life	***Taking care** through measurements and tests* ***Promoting** the bond with the unborn child by offering information and guidance* ***Cultural** challenges and preventing possible health risks*	Proctor [48] McCourt [46] Sword et al. [5] Andersson et al. [42] Aquino et al. [7] Saftner et al. [50] Goodwin et al. [45] Wright et al. [4]	Minor methodological limitations. Minor concerns about data analysis, clarity about reflexivity and clarity about ethical considerations.	Minor concerns about relevance. The primary studies support a review finding is applicable to the context. The primary studies support a review finding is applicable to the context. The ANC by midwives/nurses in different places/context and different countries.	Minor concerns regarding coherence. studies. Data reasonably consistent within and across studies.The data from the primary studies (carefully chosen) and a review finding synthesises the data good.	Minor concerns about adequacy of data. The participant and the data are presented. The whole data is rich with 16 studies and it described though some quotations.	Moderate confidence. The review finding is a reasonable representation of the phenomenon of interest. The minor methodological considerations, minor concerns about relevance, coherence and adequacy of data.
Promoting the wellbeing and health of the family today and in the future	***Providing** and sharing information and support through parenting education* ***Seeing** the importance of family members and networks and accepting them in antenatal care* ***Helping** parents connect with other people and get peer support*	Ahlden et al. [20] Sword et al. [5] Dalton et al. [43] Andersson et al. [42] Rominow et al. [49] Saftner et al. [50] Baron et al. [6] Goodwin et al. [45] Wright et al. [4]	Minor methodological limitations. Minor concerns about rigours of the data analysis, clarity about ethical considerations and relationship between researcher and participants.	Minor concerns about relevance. The primary studies support a review finding is applicable to the context. The ANC by midwives/nurses in different places/context and different countries.	Minor concerns regarding coherence. Data reasonably consistent within and across studies. The data from the primary studies (carefully chosen) and a review finding synthesises the data good.	Minor concerns about adequacy of data. The participant and the data are presented. The whole data is rich with 16 studies and it described though some quotations.	Moderate confidence. The review finding is a reasonable representation of the phenomenon of interest. The minor methodological considerations, minor concerns about relevance, coherence and adequacy of data.

Definitions of levels of confidence from the CERQual evaluation: high confidence: it is highly likely that the review finding is a reasonable representation of the phenomenon of interest. Moderate confidence: it is likely that the review finding is a reasonable representation of the phenomenon of interest. Low confidence: it is possible that the review finding is a reasonable representation of the phenomenon of interest. Very low confidence: it is not clear whether the review finding is a reasonable representation of the phenomenon of interest.
